# *In vitro* assessment of toothbrushing effects on orthodontic bracket debonding: comparing stainless steel and ceramic brackets with two adhesive systems

**DOI:** 10.3389/fdmed.2025.1722055

**Published:** 2025-12-05

**Authors:** Mahrukh Javed, Mehmood Asghar, Zainab Qasim Butt, Saood Khan Lodhi, Shahzad Ahmad, Muhammad Amber Fareed, Muhammad Kaleem

**Affiliations:** 1 Department of Basic Dental Sciences, Army Medical College, National University of Medical Sciences (NUMS), Rawalpindi, Pakistan; 2 FMH College of Medicine and Dentistry, Lahore, Pakistan; 3 Faculty of Medicine and Health Sciences, The University of Buckingham, Buckingham, United Kingdom; 4 Clinical Sciences Department, College of Dentistry, Ajman University, Ajman, United Arab Emirates; 5 Center of Medical and Bio-allied Health Sciences Research, Ajman University, Ajman, United Arab Emirates

**Keywords:** bonding agent, debonding, orthodontic adhesives, orthodontic brackets, toothbrush simulator, shear bond strength

## Abstract

**Background:**

This study aimed to compare the bracket failure of metal and ceramic orthodontic brackets bonded with two different adhesives using the conventional shear bond test and a custom-made toothbrush simulator.

**Methods:**

Forty-eight bovine teeth were extracted, bracketed, pH-cycled (14 days), randomly divided into four groups: metallic brackets bonded with B&E bonding agent (MB) and OrthoVita (MO), and ceramic brackets bonded with the B&E (CB) and the OrthoVita (CO). The bracket failure was evaluated with a universal testing machine (UTM) and a custom-made toothbrush simulator (TBS) (*n* = 6). Similarly, the amount of adhesive remaining on the bracket following debonding was evaluated using the Adhesive Remnant Index (ARI). Statistical Package for Social Sciences (SPSS) version 22 was used for statistical analysis. A two-way ANOVA was used for shear bond testing, while Fishers Exact test was used for the comparison of toothbrush simulator results.

**Results:**

The mean values of shear bond strengths (± SE) were MB 58.32 ± 4.48 MPa, MO 22.57 ± 5.64 MPa, CB 27.36 ± 2.66 MPa, and lastly, CO 12.54 ± 3.64 MPa (*p* < 0.001). In the case of toothbrush simulation, all the samples remained intact in the group MB following 75,000 cycles. In group MO: two samples debonded < 10,000 cycles, four remained intact. In CB, three samples debonded < 10,000 cycles, one debonded < 20,000–30,000 cycles, and only two samples remained intact. In the CO group four samples debonded < 10,000 cycles, while two samples remained intact.

**Conclusion:**

Metallic brackets and B&E Universal adhesive showed superior results. MB proved to have the strongest bonding comparatively.

## 1 Introduction

Malocclusion refers to a deviation from normal dental occlusion. It can be characterized by variation in tooth size, alignment and position (1, 2).As per the World Health Organization (WHO), malocclusion ranks third among oral health diseases (3), highlighting its significant impact globally (4). It can lead to various oral health problems, including dental caries and periodontitis. It can also affect the social well-being of the patient (5, 6).

There are primarily two treatment modalities for the treatment of malocclusion; removable appliances and fixed orthodontic appliances (7). Fixed orthodontic treatment employs wires and brackets and is a prevalent method for achieving optimal dental alignment and occlusion due to its efficacy in facilitating controlled tooth movement. Nonetheless, this approach is characterized by a prolonged therapy duration, typically spanning 2–3 years (8).

Bracket debonding during fixed orthodontic treatment is a common practice that unexpectedly delays the treatment duration (9, 10). The type, design and material of the bracket used greatly impact the bond failure (11, 12). The external forces and the type of adhesive used may also have a significant impact on the debonding of the orthodontic brackets (13). These factors not only prolong the treatment plan, but they also increase the chair-side time and cost which may alter the patient compliance, which is of utmost importance during an orthodontic treatment (14).

Conventionally, the bracket failure of orthodontic brackets is evaluated by using mechanical tests such as shear bond testing and dynamic fatigue tests. In the case of shear bond testing, a universal testing machine (UTM) is used which exerts a perpetually increasing true shearing or a tensile load on the specimens to debond the brackets (15). However, Despite its accuracy and precision, the UTM cannot fully mimic the clinical situation (16), since orthodontic brackets face all types of forces in all directions inside the oral cavity while the force applied by the UTM is mainly unidirectional (17). Similarly, dynamic fatigue testing, though clinically more relevant, is used routinely due to being labor-intensive and time-consuming.

One of the factors that could lead to orthodontic bracket debonding is toothbrushing (18, 19). Hence, it seems reasonable to test the resistance to debonding of brackets in a simulated environment that mimics toothbrushing. Such toothbrush simulators, in which a toothbrush head moves linearly on the surface of the tested samples, have previously been used extensively to test the wear of teeth (20–22). Similarly, a previous study also compared the effect of automated toothbrushing simulation using manual and powered toothbrushes on bond strength of orthodontic brackets (19). However, to the best of the authors’ knowledge, no study has yet compared the debonding behavior of metal and ceramic brackets using two different adhesives, following automated toothbrush simulation.

A toothbrush simulator could offer a convenient, inexpensive, and reliable option for evaluating the bracket failure of orthodontic brackets and help clinicians make evidence-based decisions to reduce treatment duration with enhanced precision. Hence, this study aims to assess the influence of toothbrushing on the debonding of orthodontic brackets (metallic and ceramic) and two commercially available adhesives using a custom-designed toothbrush simulator. The Rationale of this study is to evaluate bracket failure. One of the most frequent setbacks in orthodontic treatment is bracket debonding, which can interrupt tooth movement, extend treatment time, and cause inconvenience for both patients and clinicians. The strength of the bracket–tooth bond depends on several factors, including the type of bracket, the adhesive material, and the daily stresses applied during toothbrushing. Since brushing is a routine and necessary habit, its repeated action may gradually weaken the bond over time. Stainless steel and ceramic brackets differ in how their surfaces interact with adhesives, and different bonding agents vary in their resistance to wear.

By testing these variables under simulated brushing conditions, this study aims to identify which bracket–adhesive combinations perform best. In a clinical context, this knowledge can help reduce unexpected bracket failures, shorten treatment duration, minimize emergency appointments, and ultimately improve the overall orthodontic experience for patients.

## 2 Materials and methods

This study was approved by the institutional Ethical Review Committee (ERC/ID/370).

### 2.1 Sample collection and preparation

A total of 48 permanent bovine mandibular incisors were extracted from cows of age 4–5 years. After cleaning they were inspected for cracks under an optical microscope (Optika, B-500 Met, Italy) at magnification of 10×. The samples were disinfected with thymol (Merck, Germany) and stored in formalin (Merck, Germany). Only those teeth which were free from cracks were used for further testing. The samples were distributed to different groups via simple random samples. Each tooth was allocated a number (1–48) and the numbers were randomly distributed in four groups through MS Excel.

Sample size estimation was performed using G Power software (version 3.1.9.7). A t-test comparing the mean differences between two independent groups was performed (control and experimental) to evaluate the sample size. Mean shear bond strength values (±SD) of the control (7.22 ± 1.15 MPa) and the experimental group with the highest shear bond strenght (7.88 ± 1.61 MPa) from a previous study by Malik et al. was used (23).

After inputting the means ± SD into the software, it was shown that to obtain a power of 95%, the minimum sample size required was 8 for two groups (*n* = 4). However, a sample size of *n* = 6 was chosen. Since the current study had four groups, a total of 48 specimens were used.

**Figure 1:**
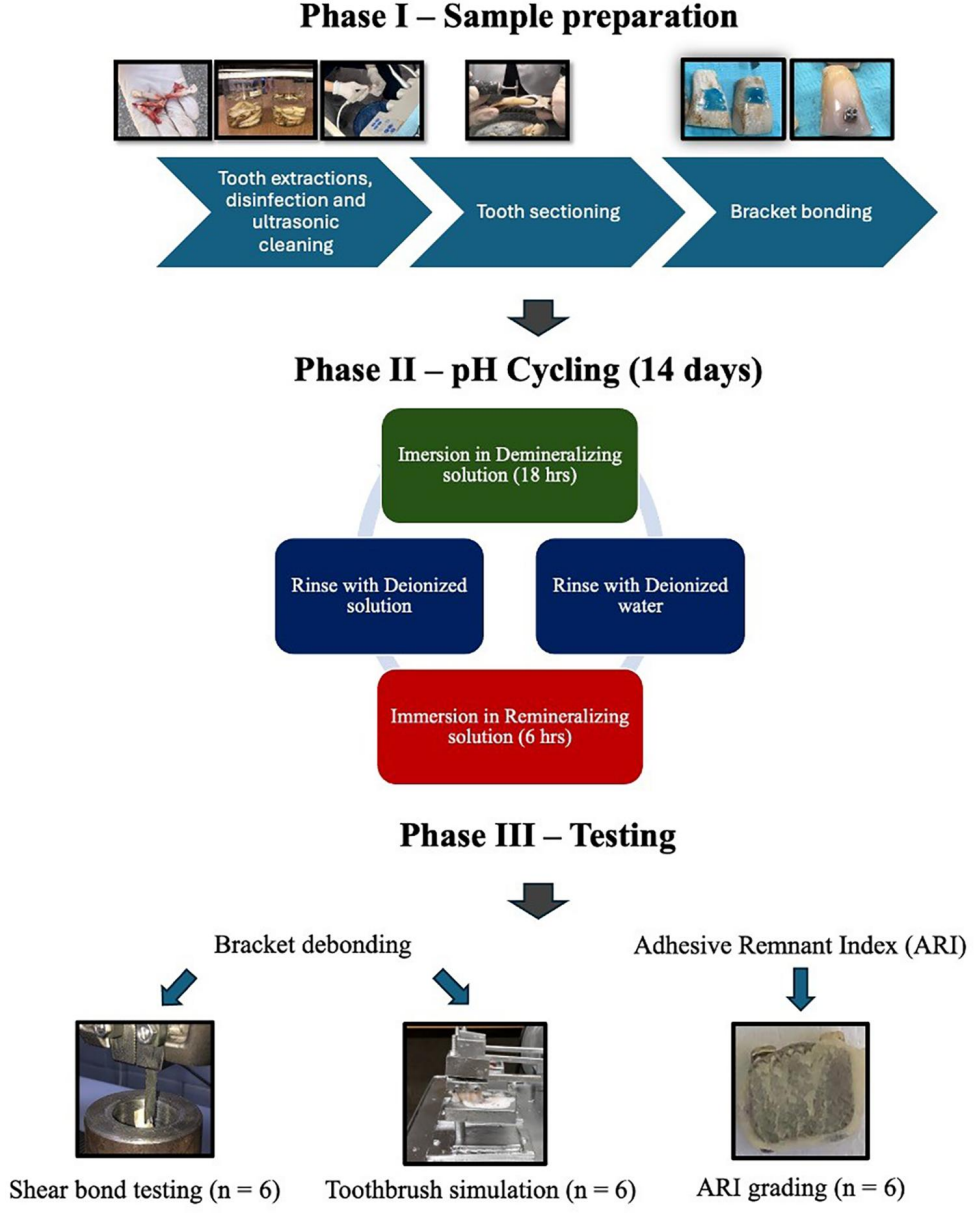
Schematic illustration showing sample preparation and testing.

A schematic representation of the phases involved in sample preparation till testing are shown in Figure 1.

### 2.2 Bracket placement

Two different types of orthodontic brackets; Metallic (MBT Mico one classic brackets, SIA Orthodontic Manufacturer, Italy) and Ceramic (MBT Mico one classic brackets, SIA Orthodontic Manufacturer, Italy) were used. Along with the orthodontic brackets, two different types of adhesives with a 3-step bonding procedure were used: B&E Universal adhesive (BNE, Korea) and OrthoVita (O&S Dental, USA). The composition of the bonding agents is given in Table 1.

**Table 1:** Sample grouping used in this study.

Group	Bonding agent	Bracket type
MB	B&E Universal adhesive (Korean)	Metallic MBT (Mico one classic brackets)
CB	B&E Universal adhesive (Korean)	Ceramic MBT (Mico one classic brackets)
MO	OrthoVita (USA).	Metallic MBT (Mico one classic brackets)
CO	OrthoVita (USA).	Ceramic MBT (Mico one classic brackets)

The bracket bonding was done according to the manufacturer's instructions. The buccal surfaces of all teeth were etched with 37% phosphoric acid for 30 s, it was followed by rinsing with a water spray for 10 s, and gently air-dried. The brackets were placed on the buccal surface of the tooth and pressed firmly into place. Excess material was removed, and the bracket was cured using curing light for 15 s at a 90˚ angle at a minimum distance to encourage maximum depth of cure.

The samples were grouped according to the bracket material and the bonding agents. Four groups were made and were labeled as shown in Table 1.

### 2.3 pH cycling

pH cycling was done to mimic the pH changes in the oral cavity. The composition of the demineralizing and re-mineralizing solutions is given in Table 2 week (24). Fresh solutions were prepared every four days and to avoid bacterial growth, a few crystals of thymol were added in both solutions.

**Table 2:** Composition of demineralization and remineralization solutions.

Chemicals	Demineralizing solution (pH 4.4)	Remineralizing solution (pH 7)
Calcium chloride	2.2 mM (0.244 g)	1.5 mM (0.166 g)
Sodium phosphate	2.2 mM (0.36 g)	0.9 mM (0.15 g)
Acetic acid	0.05 M (3 g/1.42 mL)	
Potassium hydroxide	1 M Fewer drops to adjust pH	
Potassium CHLORIDE		0.15 M Few drops to adjust pH to 7.0

Tooth samples were immersed in 4 mL demineralizing solution for 6 h. Afterwards, each sample was rinsed with deionized water and immersed in a 4 mL solution of the remineralizing agent for 18 h. This was done at room temperature of 37˚C and was followed for 14 days. After completing the 14-day cycle the samples were immersed in deionized water for 24 h. to completely wash off the solutions (25).

### 2.4 Bracket debonding

#### 2.4.1 Shear bond testing

**Figure 2:**
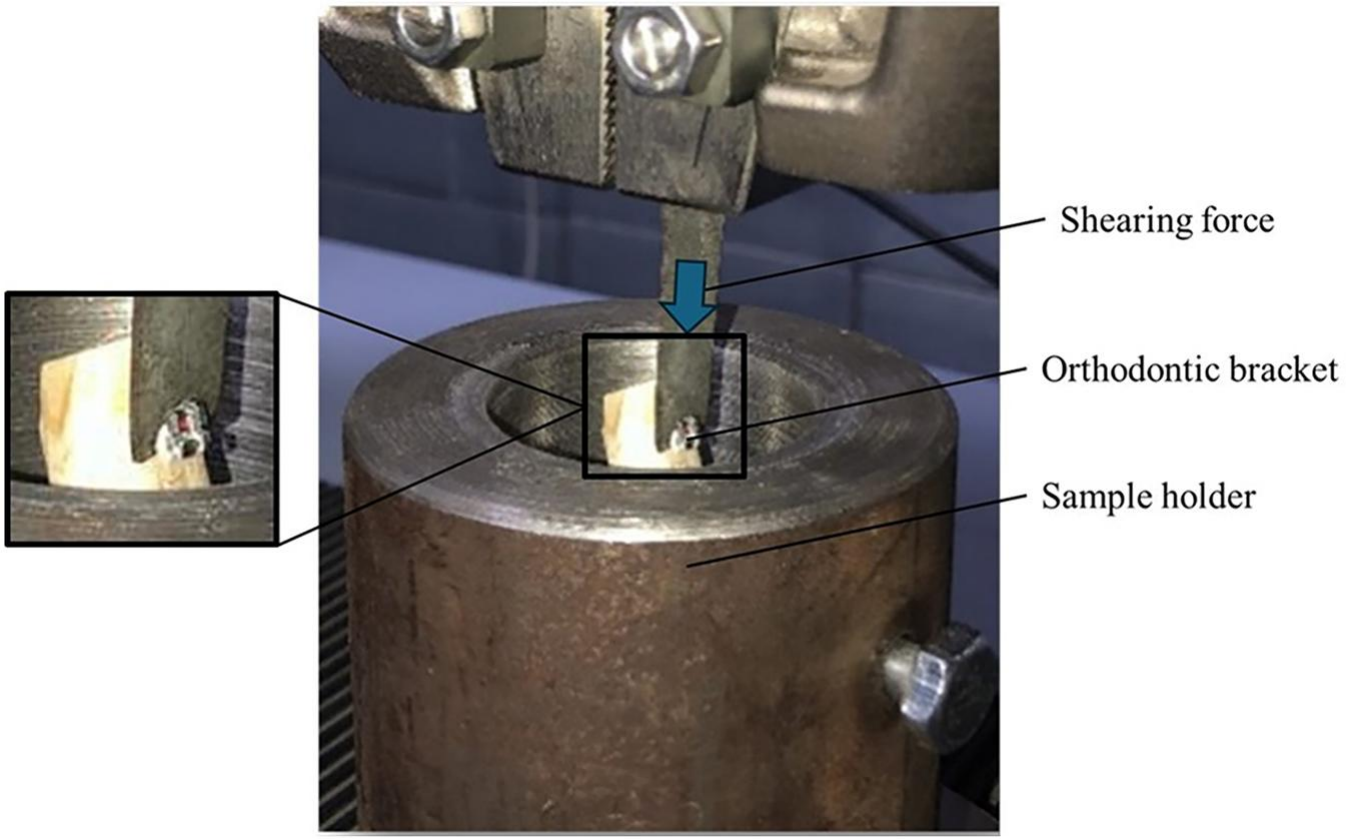
Custom made jig with test load.

Shear bond testing (*n* = 6) was performed using a Universal Testing Machine (UTM) (Walter + bai AG, Switzerland), with Load cell of 50kN and speed 1 mm/min, according to the guidelines of ISO 11405 (26). The sample size was selected based on a previously published study (23). A custom-made jig was designed to hold the tooth specimens and prevent unwanted movement during force application (Figure 2).

#### 2.4.2 Toothbrush simulation

**Figure 3:**
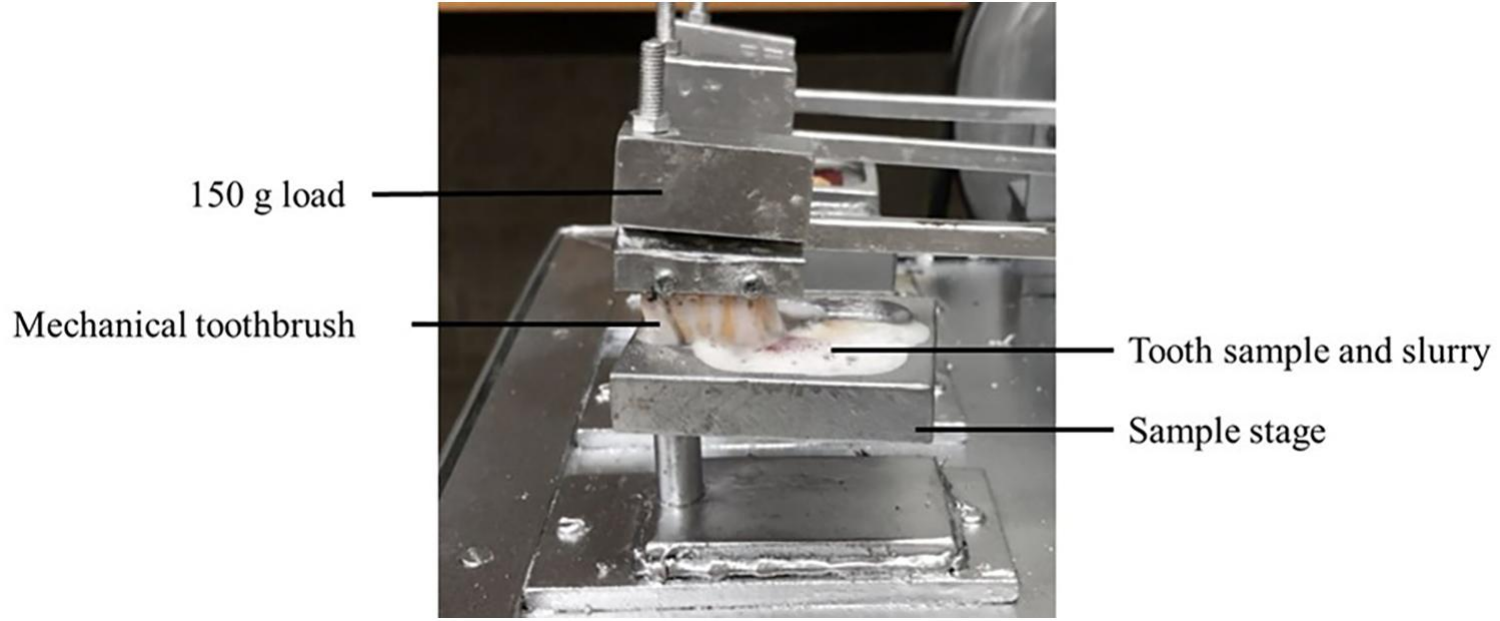
Custom made toothbrush simulator.

Toothbrush simulation was done using a custom-made toothbrush simulator (Figure 3). A custom-made toothbrushing simulator was used for this study, the details of which have been discussed in a previous study (27). Briefly, the simulator comprised a rectangular metal base supporting a horizontal shaft rotating at a fixed RPM of 87 and elevated above six sample holders. The shaft was driven by a DC motor (DC 12, China) operating a voltage of 15 Volts and a current of 4.33 Amperes. The shaft carried eccentric cams or lobes that simulated toothbrushing motion. Each cam contained a station below, where toothbrush heads or bristle simulators were mounted to mimic toothbrushing.

Six Samples were fixed in their respective holders using impression compound. Over each tooth sample, a soft toothbrush (Colgate-Palmolive, USA) was screwed such that it aligned perfectly and covered the bonded tooth surface during its to-and-fro motion. A constant load of 150 g was applied on each sample at a constant speed of 87 cycles per minute. An adhesive slurry with ratio 1:2 (toothpaste: water) was used in each toothbrush simulation as recommended by ISO 110405 (26). The number of toothbrush cycles which debonded each orthodontic bracket were evaluated. The toothbrush simulation was discontinued at a maximum of 75,000 cycles.

### 2.5 Adhesive remnant index

The amount of adhesive left on the tooth surface following debonding of the specimens was measured using the adhesive remnant index. The following criterion for the evaluation of ARI was used (Table 3) (28).

**Table 3:** Criteria of evaluating ARI (guzman et al., 2013).

Score	Evaluation
1	No adhesive left on the tooth
2	less than half of the adhesive was left on the tooth
3	half adhesive found on the tooth, half on the adhesive
4	Less than half adhesive on the bracket
5	All adhesives on the tooth

### 2.6 Statistical analysis

Data was entered and analyzed using Statistical Package for Social Sciences (SPSS) version 21. Means and standard deviations were calculated for qualitative variable e.g., SBS frequencies and percentages were reported for qualitative variables such as debonding through toothbrush simulator. A Fisher's exact test was used to find differences of debonding between groups. A two-way independent ANOVA test was used to compare differences in SBS between groups and *post-hoc* analysis was performed using Tukey test. A *p-*value of < 0.05 was considered statistically significant.

## 3 Results

### 3.1 Shear bond test

**Figure 4:**
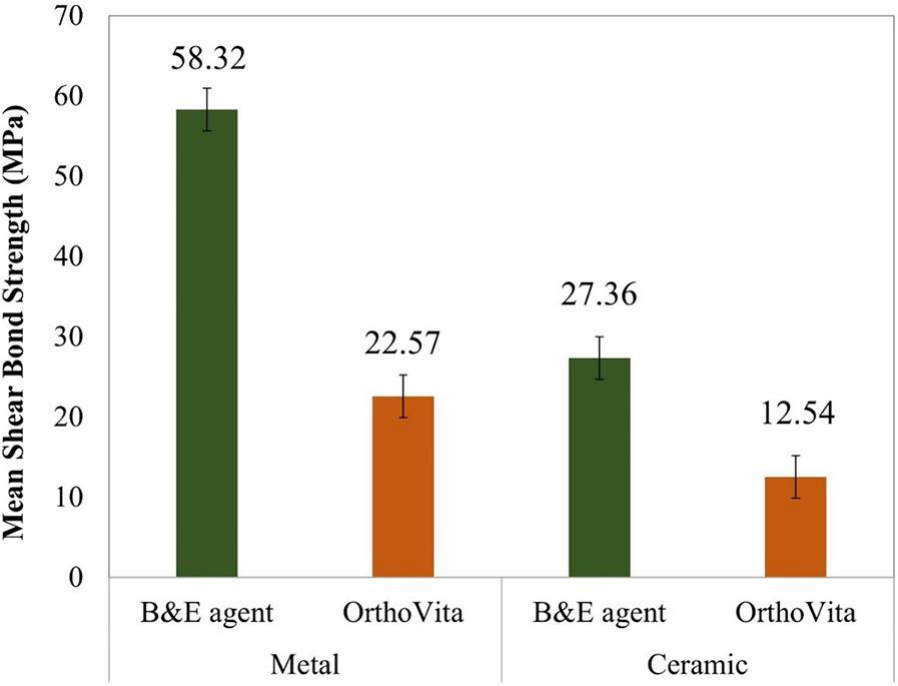
Graphical representation of results of shear bond strength.

The mean SBS values (±SE) for all the experimental groups are shown in Figure 4. MB had a mean shear bond strength value of 58.32 ± 4.48 MPa. While MO had mean SBS value of 22.57 ± 5.64 MPa. CB had mean SBS value of 27.36 ± 10.24 MPa and CO the lowest mean SBS of 12.54 ± 3.639 MPa (*p* < 0.001).

One-way ANOVA tests showed significant difference between the mean SBS when compared between the groups (bracket and adhesive type). Furthermore, *post-hoc* Tukey test showed significant differences within all the groups, when compared upon bracket or adhesive type.

### 3.2 Bracket debonding

**Figure 5:**
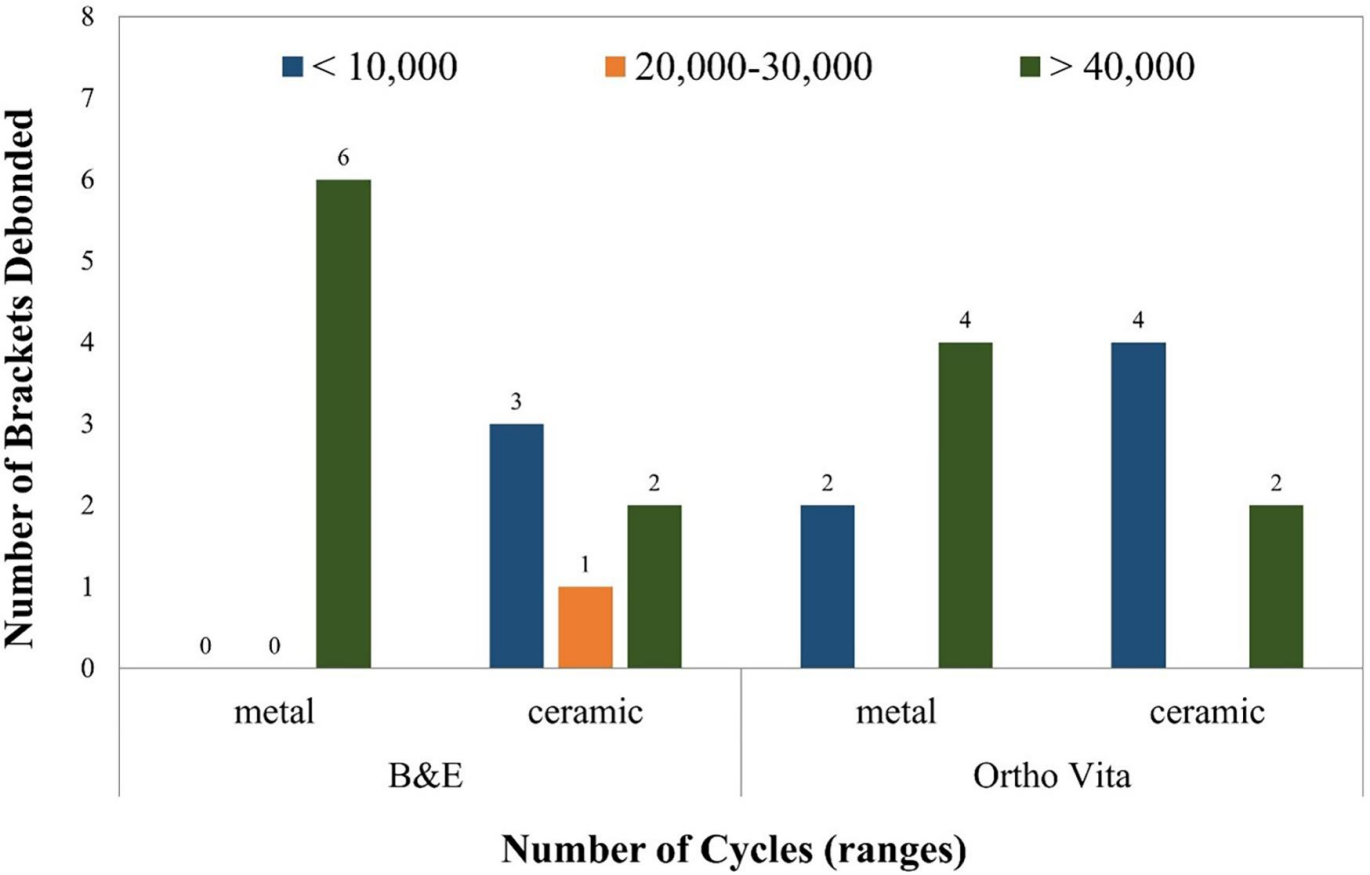
Graphical representation showing the number of cycles and the brackets debonded.

The results of bracket debonding using the toothbrush simulator are given in Figure 5. All the six samples in group MB remained intact, beyond 40,000 cycles. Three MO samples debonded within 10,000 cycles, one debonded within the second range while two remained intact. Two CB samples debonded within 10,000 cycles, however remaining four remained intact. CO showed debonding of 4 samples within 10,000 cycles while only 2 remained intact.

The results of the Fisher's exact test indicated a non-significant association between the pattern of metal and ceramic brackets debonding with either adhesive material (*p* > 0.061). However, there was a significant overall difference between when the debonding of the brackets were compared individually (*p* < 0.036).

### 3.3 Adhesive remnant index

**Figure 6:**
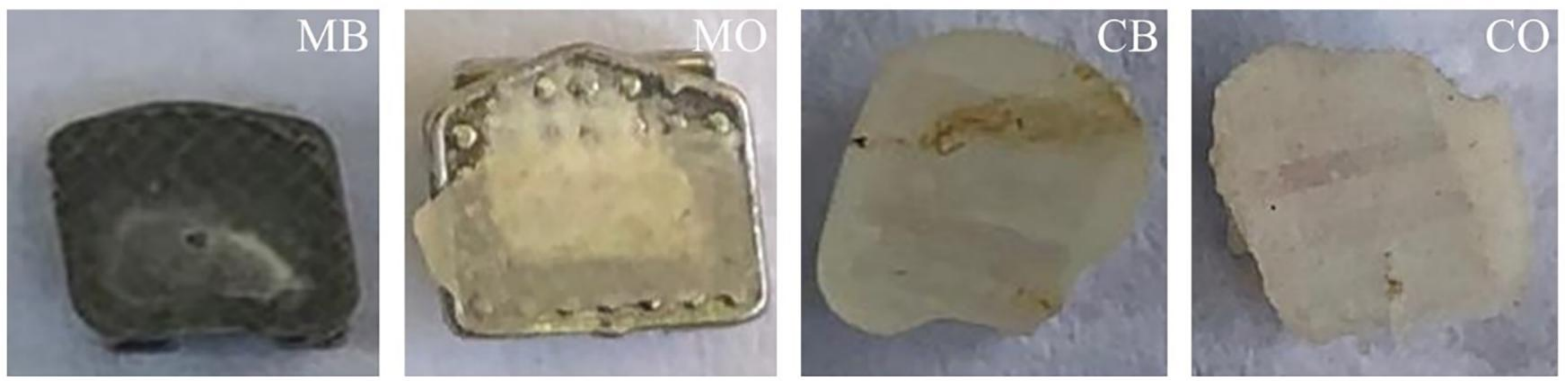
Experimental groups post debonding of the brackets.

In group MB, the predominant failure was at the tooth-adhesive interface. Four samples (90%) out of 5 were ARI grade 4 and 5 which meant all or more than half of the adhesive remained on the tooth post debonding of the bracket (Figure 6).

**Figure 7:**
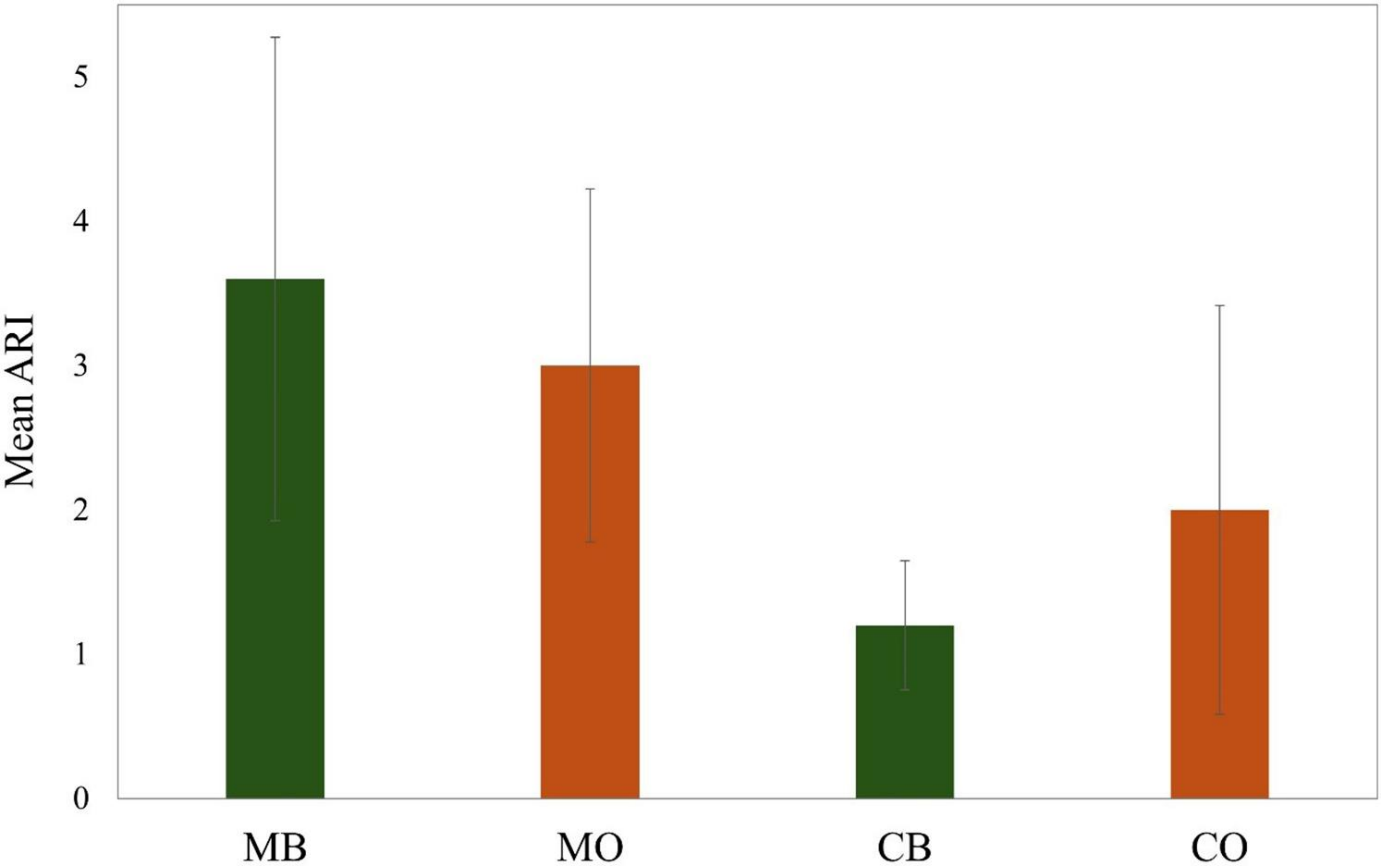
Graphical representation of ARI of the experimental groups.

For MO, three samples (60%) had grade 3 and two samples had grade 4 ARI. For CB, three samples (60%) out of five were grade 1 which meant no adhesive on the tooth after debonding. One sample was grade 3 and one was grade 4. For CO, three samples (60%) out of five were grade 1 and 2 which meant little, or no adhesive left on the tooth post-debonding. However, the remaining two samples were grade 3 which meant in these samples half the adhesive was found on the bracket and half was on the tooth. Figure 7 shows the mean ARI of the four groups.

## 4 Discussion

During orthodontic treatment, it is important to be sure that the bracket remains bonded to the tooth surface and is strong enough to endure the pressure applied during the treatment. Other factors to consider are easy removal and no enamel surface damage when debonded at the end of the procedure (29). The current study evaluated and compared the debonding pattern of two types of orthodontic brackets and adhesives using the conventional shear bond testing and a custom-made toothbrush simulator.

The results of shear bond testing in the current study followed the trend documented by Pinho et al. (30), which demonstrated that he material used for bracket fabrication played an integral role in evaluating the bond strength of the orthodontic brackets. The study showed that stainless steel brackets had a better mean bond strength value (8.9 ± 3.1 MPa) compared to the mean value of ceramic brackets (4.7 ± 1.5 MPa).

The difference observed in the shear bond strength values of the study by Pinho et al. (30) and the current study could be due to the use of bovine teeth in place of human teeth. This is because the bovine teeth form a rougher surface when etched which results in better bonding. Oval hydroxyapatite crystals present in the bovine teeth also contribute to increased bonding as they are more compact compared to the spherical crystals present in the human teeth. These factors may lead to a stronger bonding hence higher values of SBS (31).

Other possible reasons for higher bond strength values may be due to the relatively flatter enamel surfaces of bovine teeth compared to the small, curved surfaces of human teeth. This provided better adhesion and uniform thickness of the bonding agents experimented in the following study.

The results of the toothbrush simulation in this study suggested that metallic brackets showed better bonding compared to ceramic brackets and B&E adhesive had a stronger bonding compared to OrthoVita. However, higher debondings were seen in groups where ceramic brackets were used. These results are in accordance with the findings of a previous study that performed bracket debonding on human premolar teeth using pliers mounted on a testing machine (32). The authors concluded that metallic brackets had a significantly higher value of mean bond strength (30.73 ± 4.80 MPa) compared to the mean bond strength value of ceramic brackets (13.87 ± 3.87 MPa).

Similarly, Fernandez and Canut (33) concluded in their study that metallic brackets had a significantly higher tensile bond strength compared to that of ceramic brackets. Furthermore, Pont et al. (34) suggested that the bracket debonding could be affected by the type of surface trearment done on the surface of the enamel prior bracket bonding. These agents included phosphoric acid, self-etching primers and polyacrylic acid.

When comparing the bond strength of the bonding agents, B&E adhesive showed superior results compared to OrthoVita especially when used with metallic brackets. The two types of bonding agents used had different compositions. The most significant difference in composition was the different types of base monomer used for each adhesive. The type of base monomer used plays a significant role in determining the properties of composites. B&E adhesive comprised 30%–60% of urethane dimethacrylate (UDMA) while OrthoVita was incorporated with 26.02% of bisphenol-A-diglycidylmetacrylate (BisGMA), according to their manufacturers. Both base monomers have different properties.

Bis-GMA is a reaction product of bisphenol A and glycidyl ester methacrylate and is a main element of most of the dental adhesives currently used (35). It has reduced chain mobility which results in a low polymerization rate. Additionally BisGMA is highly viscous and has a hydrophobic property (36). These properties of BisGMA may have compromised the bond strength of OrthoVita in comparison to B&E.

However, B&E bonding agent has the incorporation of UDMA in combination with BisGMA. UDMA is a dimethacrylate monomer which has a low molecular weight and viscosity compared to BisGMA (37). Other advantages of UDMA over BisGMA are lower water sorption and greater toughness (38). UDMA also acts as a hydrogen donor improving the polymerization rate of the adhesive. These properties result in an increase in the amount of filler content resulting in an improved polymerization rate and a higher bond strength (37).

Fillers are also an integral component of dental adhesives. They are added to improve the bond strength of the material and to increase stiffness. It also reduces the dimensional changes of the material resulting in easy handling of the material (38). Fillers also reduce the polymerization shrinkage increasing the wear resistance of the materials (39). Despite of several advantages high concentration of filler content can have its own drawbacks. These drawbacks may include the development of air bubbles in highly filled resins used for bracket bonding. These air bubbles may interfere in the bonding of the brackets resulting in failure of the bond (40). This may be another possible reason for poor bond strength of OrthoVita as it had a higher concentration of filler content in it.

The summarized result of ARI suggests that the bond failure of ceramic groups occurred towards the tooth-adhesive interface and the bond failure of metallic groups occurred mainly at the bracket-adhesive interface. However, some samples experienced debonding within the adhesive.

Similar trend was seen in a study which suggested that stainless steel brackets fail mainly at the bracket-adhesive interface due to weak bonding. Possible reasoning for this may be due to incomplete polymerization at the bracket-adhesive interface because of the limited reach of polymerizing light under the bracket. However, after a period of 24 months, the composite got completely polymerized resulting in strong adhesive forces (41).

Joseph and Rossouw (42) showed that ceramic brackets fail at the enamel side of the bond unlike stainless steel brackets. This may be due to several factors such as poor ductility of the ceramic brackets and higher bond strength of the ceramic brackets to the adhesive (43).

This study presents several limitations that warrant consideration. Firstly, thermal cycling, which simulates the fluctuating temperature conditions of the oral cavity, was not conducted prior to testing, potentially limiting the applicability of the findings to real-world scenarios. Additionally, the evaluation of bracket debonding did not involve the application of orthodontic wires, which are commonly present in clinical settings and could influence the bonding strength. Moreover, the use of bovine teeth instead of human teeth for the *in vitro* study may introduce differences in enamel composition and structure, impacting the generalizability of the results. Furthermore, the absence of animal models for *in vivo* assessment of debonding procedures limits the extrapolation of the findings to clinical practice.

## 5 Conclusion

Despite these limitations, the study provides valuable insights. It concludes that a toothbrush simulator exhibits a similar trend of bracket debonding as conventional shear bond testing method. Additionally, the study highlights that metallic brackets demonstrate superior bonding strengths compared to ceramic brackets, suggesting a preference for metallic brackets in orthodontic practice. Notably, ceramic brackets exhibit weaker bonding unless chemical treatment is applied post-etching. Lastly, the study indicates that commercially available B&E adhesive outperforms OrthoVit adhesive in terms of adhesive bonding strength. These conclusions underscore the importance of considering various factors, including bracket material and adhesive type, in orthodontic treatment planning to ensure optimal outcomes. These findings will enable clinicians to choose the best adhesive-bracket combination for treating orthodontic patients, which will directly help minimize brackets failures, which will help decrease treatment time and cost and improve treatment quality.

## 6 Clinical implications

This study evaluated the effect of two different types of bonding agents and orthodontic brackets on the debonding behavior under simulated conditions. The findings of this study will enable clinicians to choose the best adhesive-bracket combination for treating orthodontic patients, which will directly help minimize brackets failures, which will help decrease treatment time and cost and improve treatment quality.

## Data Availability

The raw data supporting the conclusions of this article will be made available by the authors, without undue reservation.
